# Assessing how information is packaged in rapid reviews for policy-makers and other stakeholders: a cross-sectional study

**DOI:** 10.1186/s12961-020-00624-7

**Published:** 2020-09-29

**Authors:** Chantelle Garritty, Candyce Hamel, Mona Hersi, Claire Butler, Zarah Monfaredi, Adrienne Stevens, Barbara Nussbaumer-Streit, Wei Cheng, David Moher

**Affiliations:** 1 Knowledge Synthesis Group, Clinical Epidemiology Program, Ottawa Hospital Research Institute, The Ottawa Hospital, General Campus, CPCR Building, 501 Smyth Rd, Box 201B, Ottawa, ON K1H 8L6 Canada; 2 TRIBE Graduate Program, University of Split School of Medicine, Split, Croatia; 3 Cochrane Austria, Danube University Krems, Krems a.d. Donau, Austria; 4 School of Epidemiology and Public Health, University of Ottawa, Ottawa, Canada

**Keywords:** rapid reviews, health policy, health systems, decision-making, evidence synthesis

## Abstract

**Background:**

Rapid reviews (RRs) are useful products to healthcare policy-makers and other stakeholders, who require timely evidence. Therefore, it is important to assess how well RRs convey useful information in a format that is easy to understand so that decision-makers can make best use of evidence to inform policy and practice.

**Methods:**

We assessed a diverse sample of 103 RRs against the BRIDGE criteria, originally developed for communicating clearly to support healthcare policy-making. We modified the criteria to increase assessability and to align with RRs. We identified RRs from key database searches and through searching organisations known to produce RRs. We assessed each RR on 26 factors (e.g. organisation of information, lay language use). Results were descriptively analysed. Further, we explored differences between RRs published in journals and those published elsewhere.

**Results:**

Certain criteria were well covered across the RRs (e.g. all aimed to synthesise research evidence and all provided references of included studies). Further, most RRs provided detail on the problem or issue (96%; *n* = 99) and described methods to conduct the RR (91%; *n* = 94), while several addressed political or health systems contexts (61%; *n* = 63). Many RRs targeted policy-makers and key stakeholders as the intended audience (66%; *n* = 68), yet only 32% (*n* = 33) involved their tacit knowledge, while fewer (27%; *n* = 28) directly involved them reviewing the content of the RR. Only six RRs involved patient partners in the process. Only 23% (*n* = 24) of RRs were prepared in a format considered to make information easy to absorb (i.e. graded entry) and 25% (*n* = 26) provided specific key messages. Readability assessment indicated that the text of key RR sections would be hard to understand for an average reader (i.e. would require post-secondary education) and would take 42 (± 36) minutes to read.

**Conclusions:**

Overall, conformity of the RRs with the modified BRIDGE criteria was modest. By assessing RRs against these criteria, we now understand possible ways in which they could be improved to better meet the information needs of healthcare decision-makers and their potential for innovation as an information-packaging mechanism. The utility and validity of these items should be further explored.

**Protocol availability:**

The protocol, published on the Open Science Framework, is available at: osf.io/68tj7

## Background

Having ready access to relevant information to inform decision-making is vital to policy-makers who make decisions in healthcare that affect populations. Often, systematic reviews (SRs), a benchmark tool in evidence synthesis, are used to inform practice or policy [1, 2]. However, when evidence is needed to inform an emergent issue outside the traditional SR timeline of 1–2 years [3, 4], ‘rapid reviews’ (RRs) have become a practical tool to get evidence to decision-makers more quickly, often ranging from a few weeks to usually no more than 6 months [3, 5, 6]. A defining feature of RRs is the streamlining of methodological aspects of the SR process to produce information faster than most SRs [3, 5, 7, 8].

Clinically, RRs have been used to inform frontline patient care decisions [9–11], to make crucial decisions about health system responses [12–14], and to inform routine situations to improve public health [15–17]. They are also produced and used in low- and middle-income countries to support healthcare decisions [18–20]. RRs should therefore include relevant content and be designed to maximise relevancy for key stakeholders, including policy-makers, health system managers, administrators and clinicians, who are likely to use research to inform choices about the practice and delivery of care.

RRs may include summaries of SRs as well as primary studies and grey literature and have become attractive products for decision-making [21, 22]. It remains unclear, however, how well they are packaged so that evidence may be readily consumed and applied. Some studies have looked at ways to better parcel SR content and format, including ways to tailor information for clinicians, health policy-makers and health system managers by developing summaries of SRs [23–29]. Assessment of these summaries suggest that they are likely easier to understand than complete SRs by such end-users [29], who favour clear, concise summaries in simple, easy to understand language [24, 26–29]. Because RRs can take many forms and, similarly, are intended to provide a summation of evidence, knowledge on summaries of SRs may be useful for the packaging of RRs.

The BRIDGE criteria is an evidence-informed framework of building blocks of effective information-packaging to support policy-making and originated as part of a research series established to meet the needs of policy-makers and health systems managers [30]. The original BRIDGE criteria, with an emphasis on health systems research, is comprised of 11 questions across key domains designed to assess evidence products considered to be information-packaging mechanisms (e.g. a study summary, a SR summary, a compendium or grouping of summaries on a particular topic, a policy brief, or a policy dialogue report). The criteria address five specific domains, including ‘coverage’ of a health system issue or condition, in particular how topical or relevant the issue is along with its various facets, what type of knowledge the product includes (e.g. synthesised evidence, tacit knowledge and views of policy-makers and stakeholders), how and for whom it is targeted, how clearly the information is presented, and how its use is supported by end-users. According to the BRIDGE study authors, the purpose of assessing evidence products against these criteria was to encourage debate and innovation about the ways in which information is prepared and packaged for policy-makers and stakeholders as a component of an overarching knowledge-brokering approach. Given increases in the production and use of RRs, we used the BRIDGE criteria to assess a sample of RRs as a type of information-packaging mechanism. Previously applied to evidence products [30, 31], we further modified the criteria by operationalising some original items to make them more assessable and by including new criteria relevant to the context of RRs.

### Objective and research question

To date, the question of how well RRs are packaged for use in decision-making for policy-makers and other stakeholders has not been explored. Therefore, the objective of this study was to examine the extent to which RRs are a useful information-packaging mechanism based on criteria for communicating clearly to support healthcare decision-making. Our research question was: How well do rapid reviews (RRs) perform when evaluated against adapted BRIDGE criteria developed to assess information-packaging mechanisms of evidence products?

## Methods

### Study design

This was a descriptive, cross-sectional study involving a diverse sample of RR reports. The protocol for this study is available at: https://osf.io/68tj7.

Although there is no specifically endorsed definition of a *RR*, we defined it as a report where the intent is to summarise evidence for use in any form of decision-making, directly or indirectly related to a patient or to healthcare, using abbreviated and/or accelerated SR methodology to accommodate an expedited turnaround time [3, 5, 32]. We considered the ‘key stakeholders’ to be the major knowledge users in the healthcare system, comprised of policy-makers at various levels of government as well as individuals likely to use research results to make informed decisions about health policies, programmes or practices.

### Identifying RRs for inclusion (dataset)

We based our analysis on a sample of 103 RRs that included both journal-published (JP) and non-journal-published (NJP) RRs, which were identified from a parallel methods project [33]. Briefly, the JP RRs were identified by searching Ovid MEDLINE, Ovid MEDLINE In-Process and Other Non-Indexed Citations, Ovid EMBASE, Ebsco CINAHL, Proquest Educational Resources Information Center (ERIC), PsycINFO, and the Cochrane Library using search strategies that were developed in conjunction with and peer reviewed by experienced information specialists. We first completed screening of the JP literature and then conducted a grey literature search in order to identify NJP RRs. This involved reviewing the websites of 148 organisations from across five continents that produce or commission RRs as well as websites listed in CADTH’s Grey Matters checklist [34], among other sources. Because there were several hundred NJP reports identified across a mix of higher and lower RR-producing organisations, we needed an appropriate sampling strategy that took volume and product type into account knowing that some organisations produce more than one form of RR. Hence, we sampled proportionate to cluster size by organisation and RR type, using the sample size of the JP group as a guide. Given this was a descriptive, exploratory study and was therefore hypothesis generating, no formal sample size was calculated.

**Fig. 1:**
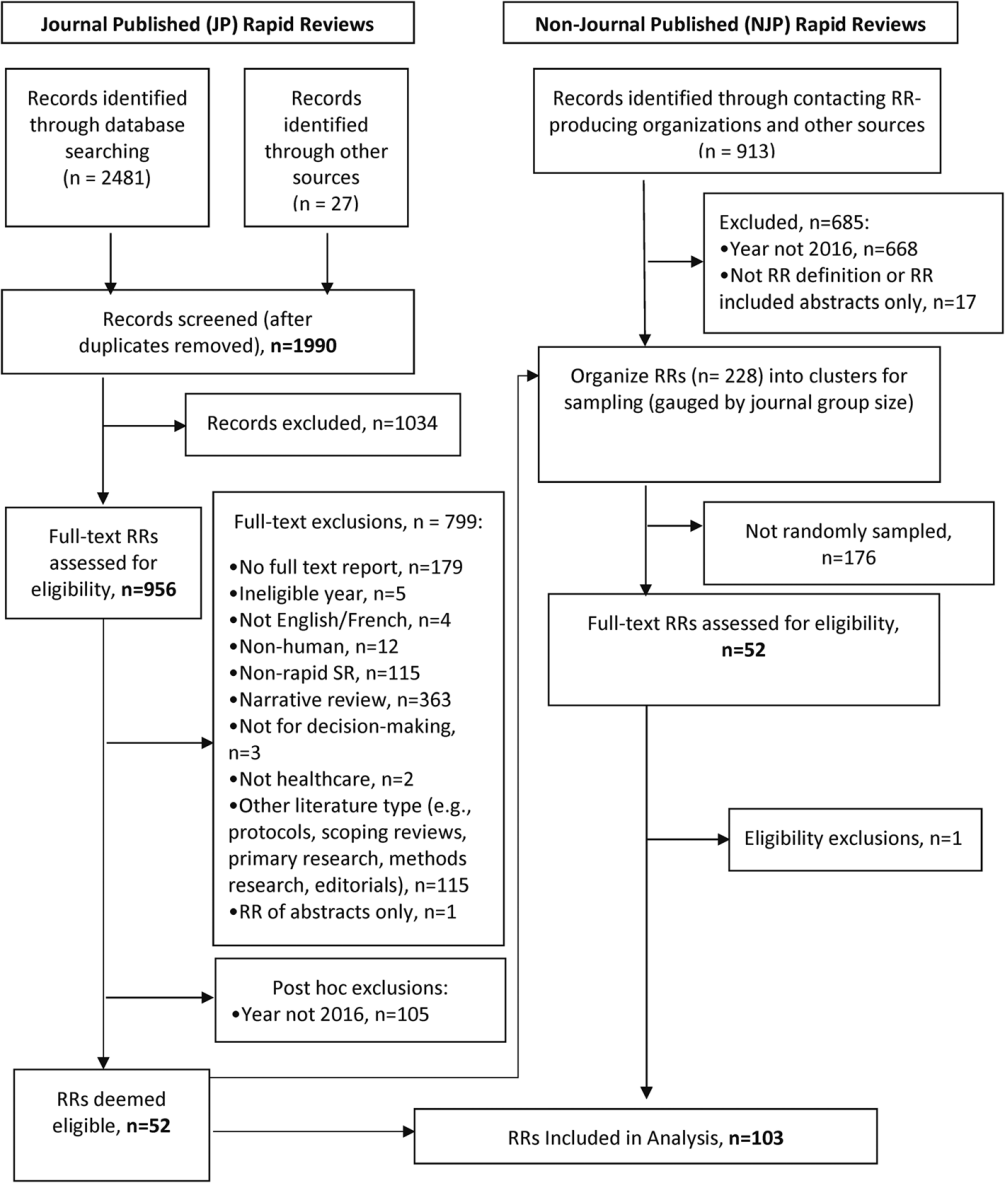
Study flow diagram. Breakdown of the number of rapid review reports identified, assessed for eligibility and included in the main sample

We assessed the eligibility of the RRs following a pilot testing of screening forms. Two reviewers independently assessed records against inclusion criteria developed a priori at title and abstract level, and then at full-text, with disagreements resolved by consensus or, if needed, by a third reviewer. Reasons for exclusion of full text reports is documented in a flow diagram (Fig. 1) that details the study selection process. We limited inclusion of RRs to those published or produced in 2016. All types of RRs related to humans and healthcare covering various topics were eligible. We did not limit by length of time it took to perform the RR, but we did exclude reports that appeared to be annotated bibliographies of relevant papers. In addition, only studies in English and French were considered for inclusion. Further details on the search strategies developed to identify the sample, eligibility criteria and the sampling frame are provided elsewhere [33].

### Applying modified criteria

Table 1 represents the original BRIDGE criteria, including the major categories covered [30], that were modified for a previously reported study [31]. Taken together, we made additional adaptations and operationalised certain items to increase the objectivity of our assessments. In addition to design and document organisation, we extended the criteria to convey broader attributes of RRs, including relevancy of content, quality of the evidence, reporting and stakeholder engagement.

**Table 1:** Adapted BRIDGE criteria

BRIDGE category [30]	Criteria [30, 31]	Adapted BRIDGE criteria for rapid reviews
I. What it covers	1. **Topical/relevant issue** from the perspective of the policy-makers with an explicit process for determining topically/relevance (e.g. periodic priority-setting exercise, rapid response service)	A. *Was the RR requested, commissioned or conducted for decision-making purposes?* ^*a*^ B. *Was the RR conducted through a rapid response service?* C. *Was the RR topic identified through a priority-setting exercise?*
	**2. Document explicitly addresses at least four or more of the following:** political and/or health system contexts, problem, options, implementation considerations, and cost implications Note: it addresses the many features of an issue, including the underlying problem(s)/objective(s), options for addressing/achieving it, and key implementation considerations (and if only some features are addressed, acknowledges the importance of the others)	D. *Does this RR address at least four or more of the following for the issue being reviewed:* • *Political and/or health system contexts* • *Problems* • *Options* • *Implementation considerations* • *Cost implications*
II. What it includes	**3. Draws on synthesised/assessed (global) research evidence** that has been assessed for its quality and local applicability	E. *Does the RR draw on synthesised/assessed, global research evidence?*
	**4. Incorporates the tacit knowledge of policy-makers/stakeholders** that has been collected in a systematic way and report in a transparent manner	F. *Does the RR incorporate tacit knowledge of policy-makers and/or stakeholders?* G. *Has the tacit knowledge been collected in a systematic way and reported in a transparent manner?*
III. For whom it is targeted	5. **Explicitly targets policy-makers/stakeholders** as the key audience Note: it targets policy-makers and stakeholders with an explicit statement about them being a key audience (not just a policy implications section)	H. *Does the RR explicitly report target policy-makers and/or stakeholders as the key audience?*
	6. **Engages policy-makers/stakeholders in reviewing the product for relevance and clarity?**	I. *Was the RR report reviewed by policy-makers and/or stakeholders (not just researchers) for relevance and clarity?*
		***Patient engagement in research*** ^***a***^ J. *Was the RR reviewed by patients/consumers for relevance and clarity?* K. *If applicable, were patients involved in any phases of the rapid review conduct? Check all that apply* o *Preparatory phase (agenda-setting, prioritisation of research topics and funding)* o *Execution phase (study design and procedures, screening, data collection, and/or data analysis)* o *Translation phase (interpretation of findings, dissemination, implementation or evaluation)*
IV. How it is packaged	7. **Organised to highlight decision-relevant information**	L. *Was the RR organised in such a way to highlight decision-relevant information? For example, are benefits, harms and costs of policy/programme options highlighted in some capacity in the report?*
	8. **Written in understandable/lay language**	M. *Was understandable, lay language used?* ^*a*^ • *SMOG score of report* • *Word count of report* • *Estimated reading time (minutes)*
	9. **Prepared in a format that makes the information easy to absorb? Is readily appreciated** (e.g. graded entry)	N. *Was the prepared in a format that makes the information easy to absorb? (e.g. graded-entry)*^*b*^
V. How its use is supported	10. **Contextualised through online commentaries/briefings** provided by policy-makers/stakeholders	O. *Was the RR contextualised through online commentaries/briefings provided by policy-makers/stakeholders?*
	11. **Brought to the attention of target audiences through email/listserv**	P. *Was the RR brought to the attention of target audiences through email, listservs or website postings* ^*a*^*?*
VI. Features and content [18]	12. **Equity considerations** discussed or implicitly considered (e.g. through topic or analysis)	Q. *Are equity considerations discussed or implicitly considered (e.g. through the topic or analysis)?* *In assessing, consider whether the rapid review addresses any of the following* [35]:^a^ 1. *Which group or settings are likely to be disadvantaged relative to the policy option being considered?* 2. *Are there reasons for differences in the relative effectiveness of the option for disadvantaged groups or settings?* 3. *Are there likely to be baseline differences across groups or settings that could influence the effectiveness of the option? Would these baseline differences mean the problem is more or less important for disadvantaged groups or settings?* 4. *What should be considered when implementing the proposed option to ensure that inequities are reduced and/or not increased?*
	13. **Recommendations provided**	R. *Did the RR provide recommendations?*
	14. **Methods described**	S. *Were the methods to conduct the RR described?*
	15. **Quality of research evidence and/or limitations outlined**	T. *Was quality assessment/risk of bias assessment of the included research evidence conducted?* U. *Were limitations of the RR approach outlined?*
	16. **Reference list provided**	V. *Was a reference list provided?*
	17. **Local applicability discussed,** including case examples to highlight how a particular policy might be adapted to local circumstances	W. *Was local applicability discussed in the RR?* X. *Were case examples included illustrating how to adapt or apply a policy or intervention locally?*
	18. **Key messages or summary points provided**	Y. *Were key messages or summary points provided in the RR? (i.e. specifically labelled in the report as such)*
	19. **How is the rapid review labelled?**^**a**^	Z. *Does the RR self-declare as ‘rapid’ (explicit phasing) in title or body?*

*RR* rapid review

^a^New criterion or item added

^b^GRADED Entry – a report structure organised to highlight decision-relevant summarised information upfront followed by more detailed information that is gradually uncovered for the reader [42, 43] versus IMRAD – the predominant format of academic journal articles (Introduction, Methods, Results, and Discussion)

Specifically, we added three further items. The first item added, in order to help assess whether the RR addressed a topical/relevant issue, was whether or not the request for RR had been reported, commissioned, or conducted for decision-making purposes (Table 1 – Criterion 1, Item A). The second item added pertained to patient engagement in the development of the RR (Table 1 – Criterion 6, Item J), and if applicable, at which stages of the process patients may have been involved. The term ‘patient’ refers to anyone who has personally lived the experience of a health issue as well as their informal caregivers, including family and friends [36]. Research has shown that individuals who are engaged in their health are more likely to achieve better health outcomes [37]. In Canada and elsewhere, a key component to patient engagement are strategies involving their participation as partners in research. Therefore, we sought to capture the extent of patient/partner involvement in our sample of RRs. The third item added was how each RR report was labelled (i.e. did the report self-declare as ‘rapid’ in its title or body?) (Table 1 – Criterion 19, Item Z) to determine how similar or varied the nomenclature used across the spectrum of RRs may be and to highlight the potential impact this may have on RRs collectively as an information product.

In addition, we also operationalised certain items with the aim to increase clarity and consistency when applying the criteria. In particular, we expanded on components that assessed if the RR was written in comprehensible or lay language (Table 1 – Criterion 8, Item M) by examining the readability and estimated reading time of the RRs based on word count. Previously, we collected data on the reading level across three key sections of each RR (i.e. abstract/summary, introduction/background and discussions/conclusions) according to the Simple Measure of Gobbledygook (SMOG) readability test, using an online calculator (https://www.learningandwork.org.uk/SMOG-calculator/smogcalc.php) to generate the SMOG scores that estimate the years of education a person needs to understand a piece of writing [38]. Evidence suggests that the SMOG is the most appropriate readability measure for assessing written health information [39]. In addition, we further examined the word count for each RR for both the main body of the report and the total word count (including references and appendices) using the Microsoft Word built-in word-count function. From this, we estimated the reading time of the RRs by dividing the total word count of each report by 200, which is the number of words on average a person is able to read per minute for comprehension [40].

In terms of item clarity, when assessing if the RR has been prepared in a format that is readily appreciated (Table 1 - Criterion 9, Item N), we provided guiding definitions of what constitutes two key format structures (i.e. IMRaD and graded entry). IMRaD is an acronym that refers to the Introduction, Methods, Results and Discussion sections of an original article and is the standard format of academic journal articles [41]. A graded entry format structure is organised differently to highlight decision-relevant, summarised information upfront followed by more detailed information that is gradually uncovered for the reader [42, 43]. Graded entry structures typically include most IMRaD components but may present them in a different order to facilitate the uptake of information. Therefore, when assessing readability (Table 1 – Criteria 8, Item M), we needed to adjust which sections to assess depending on whether the RRs adhered to a traditional publication format type (i.e. IMRaD) or more non-traditional formats (e.g. graded-entry, multicomponent report or other types of structures, including any combination of format types).

With regards to equity considerations, we provided four statements to guide assessment of this item (Table 1 – Criteria 12, Item Q) originally developed as part of a package of tools for policy-making specifically taking parity into consideration when assessing the findings of a SR [35].

Lastly, we reduced the number of double-direct item questions that originally touched upon more than one issue, yet previously allowed only for one answer. Where appropriate, we separated these items into discrete criteria to decrease ambiguity when assessing the RRs. For example, ‘quality of the research evidence and/or limitations outlined’ [31] was presented as two items in our assessment (Table 1 – Criteria 15, Items T & U). In addition, Criteria 3, 4 and 17 were similarly modified. In total, each RR was assessed against a total of 26 factors.

### Data extraction process

Prior to data extraction, we conducted a pilot extraction of five articles to ensure consistent interpretation of criteria were applied to the studies. One reviewer extracted data using pre-tested data extraction forms (available at www.osf.io/68tj7) (CG, ZM, CB). A second reviewer crosschecked all extracted data (CG, CB, CH). We gathered general study characteristics (e.g. country of corresponding author or producer, funding, time to completion, purpose or rationale for the RR conveyed) for each RR prior to applying the criteria, for which most items were coded as yes or no/not reported. We resolved disagreements through consensus by referring to the study report. Because it was our intent to evaluate each report in the same manner it was made available (packaged) for end-users, we did not follow-up with producers for further clarification. We used Reference Manager [44] to manage all citations and an online software to screen and extract eligible studies (DistillerSR by Evidence Partners) [45].

### Data analysis

Given the nature of this study, we used descriptive summary statistics to assess the RRs against each criterion. Specifically, we calculated the median and interquartile range for continuous data items and proportions for binomial items. Categorical sub-items were reported as counts within each category.

#### Exploratory analysis

Using Fisher’s exact test for binomial proportions (with odds ratio (OR) estimates based on conditional maximum likelihood method) and Welch’s *t* test for mean differences of continuous data items, we explored whether there were significant differences on items between JP and NJP RRs. All analyses were performed using Microsoft Excel and R version 3.5.3 (http://www.R-project.org/).

Although no reporting guideline exists for this type of methodology study, we completed the STROBE Statement—Checklist for cross-sectional studies to the extent possible (Additional file 1).

## Results

Amendment to the protocol – we did not include sentiment analysis as originally planned as we deemed this not to be informative to the readability of the RR documents identified. This represents a deviation from the original protocol but had no impact on the results of the study (https://osf.io/68tj7/).

### Search results

As identified from a parallel methods project [33], following the screening of 1990 JP records and 227 full-text reports produced by various RR-producing organisations, a total of 103 RRs were included (Fig. 1). Overall, we applied the modified BRIDGE criteria to 52 JP and 51 NJP RRs reports. All RRs were in English with the exception of one French JP RR.

Table 2 provides full details on the general study characteristics of the included reports. RRs were identified from a total of 15 countries, with the majority produced by Canada, followed by the United Kingdom, Australia and the United States. The 52 JP RRs were identified from 47 unique journals (across 21 distinct publishers) that were primarily speciality journals (37/52; 71%) (Additional file 2). Further, the median (interquartile range; range) journal impact factor of these RRs was 2 (1; 0.57–47.83). The 51 NJP RRs were identified from 25 unique organisations based in six different countries.

**Table 2:** General characteristics of included rapid reviews

Characteristics	All (***n*** = 103)	Journal published (***n*** = 52)	Non-journal published (***n*** = 51)
Country of corresponding author or producer, n (%)			
Canada	42 (41)	12 (23)	30 (59)
United Kingdom	21 (20)	20 (38)	1 (2)
Australia	14 (14)	4 (8)	10 (20)
United States	10 (10)	3 (6)	7 (14)
Belgium	3 (3)	2 (4)	1 (1)
Scotland	3 (3)	1 (2)	2 (4)
Italy	2 (2)	2 (4)	0
China, Denmark, Germany, Netherlands, Saudi Arabia, Spain, Sweden, Taiwan^a^	1 (1)	1 (2)	0
List of authors cited, *n* (%)	89 (86)	52 (100)	37 (73)
Reported funding, *n* (%)	67 (65)	39 (75)	28 (55)
Funding source, *n*			
External, peer-reviewed grant	8	6	2
External, non-commercial (fee for service)	47	22	25
External, commercial (fee for service)	2	2	0
Internal	1	0	1
Specified no funding received	9	9	0
Purpose or rationale for RR conveyed by the authors	63 (61)	33 (63)	30 (59)
Time to conduct the RR reported, *n* (%)	6 (6)	3 (6)	3 (6)
4 weeks	2	0	2
8 weeks	1	1	0
17 weeks	1	0	1
24 weeks	1	1	0
32 weeks	1	1	0
Main intervention, *n* (%)			
Pharmacological	17 (17)	4 (8)	13 (25)
Non-pharmacological	57 (55)	29 (56)	28 (55)
Mixed	5 (5)	1 (2)	4 (8)
Other (does not address an intervention or exposure)	24 (23)	18 (35)	6 (12)
Number of study designs included in the RRs, *n* (%)			
One	37 (36)	14 (27)	23 (45)
Two or more	66 (64)	38 (73)	28 (55)
Frequency of included study designs, *n*^b^			
Systematic reviews	40	15	25
Randomised controlled trials	41	17	24
Observations studies (cohorts, case-control, cross-sectional)	61	36	25
Other^c^	37	21	16
Unclear	40	28	12
Peer reviewed, *n* (%)	56 (54)	50 (96)^d^	6 (12)^e^
RRs publicly available, *n* (%)	86 (83)	36 (69)	50 (98)
Journal Impact Factor, median (inter-quartile range)[range]^f^	n/a	2 (1) [0.57–47.83]	n/a
Language of the RRs in English, *n* (%)	102 (99)	52 (100)	50 (98)

*RR* rapid review

^a^Per country

^b^Other may qualitative, quasi-experimental design including interrupted time series, controlled before/after, case series etc.

^c^Denotes the frequency of the included study designs

^d^Peer review confirmed if journal listed on the DOAJ or if specifically stated as a policy of the journal

^e^Non-journal-published RRs peer review status based on reporting of methods in each report and/or from available methods guidance from respective institutions

^f^Based on unique journals (*n* = 47), of which 39 reported impact factors for 2016 (Additional file 2)

### Modified BRIDGE criteria

**Fig. 2:**
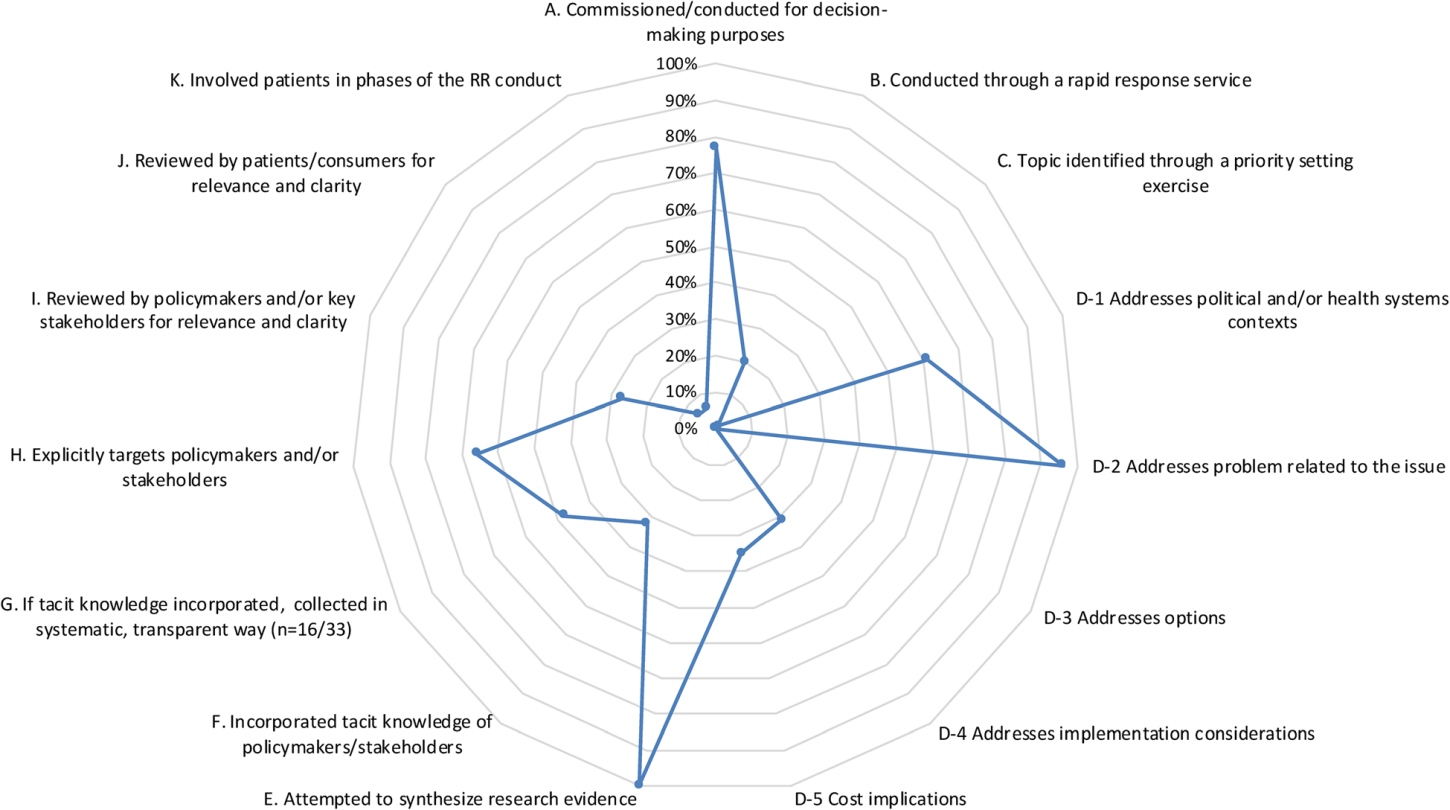
Radar chart depicting proportions of rapid reviews adequately meeting adapted BRIDGE criteria (*n* = 103) (Items A–K)

**Fig. 3:**
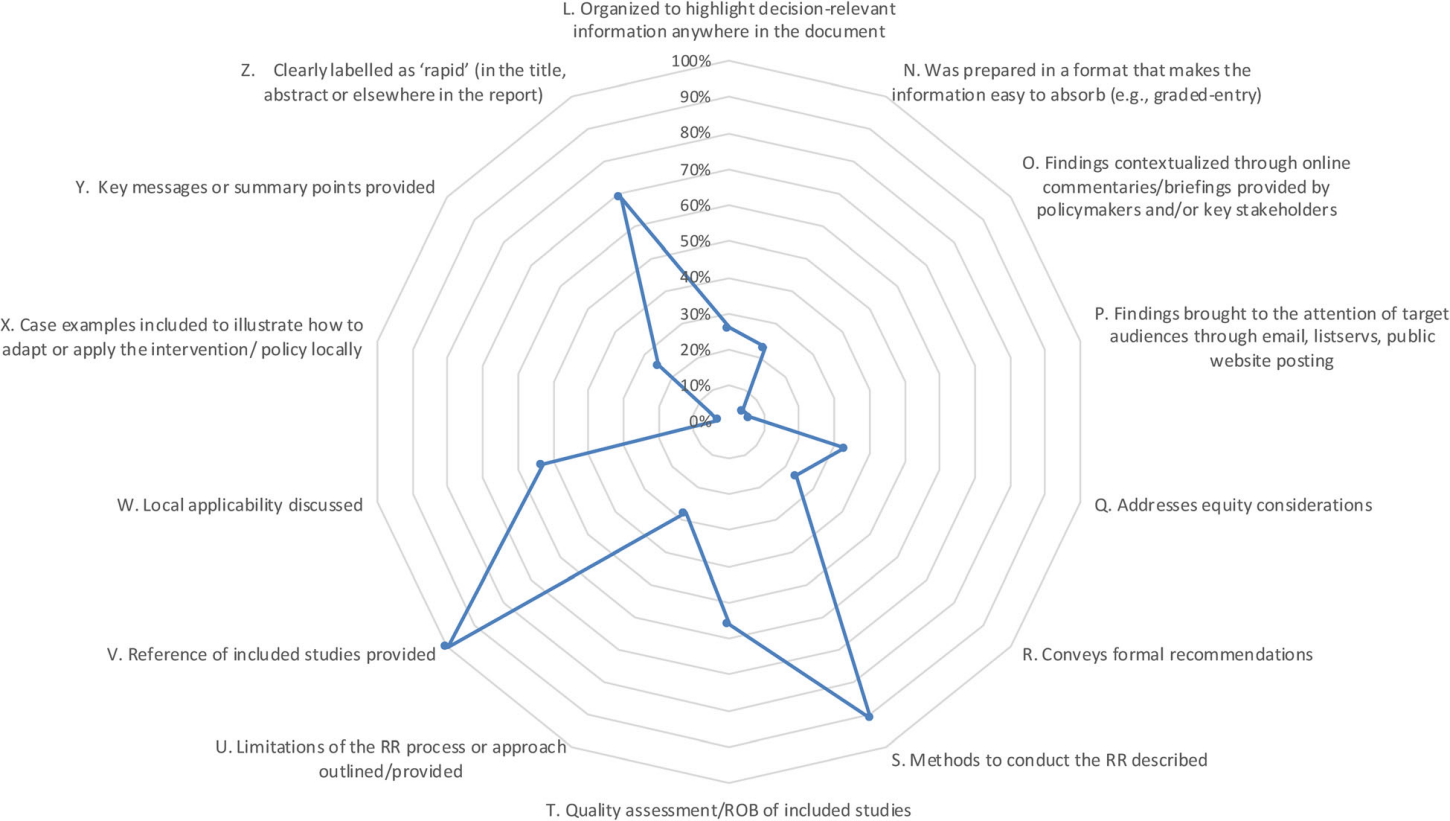
Radar chart depicting proportions of rapid reviews adequately meeting adapted BRIDGE criteria (*n* = 103) (Items L–Z)

Figures 2 and 3 show the proportion of RRs (*n* = 103) that adequately met the individual adapted BRIDGE criteria, for which yes/no responses were obtained. Full results of the adapted BRIDGE criteria as applied to our sample of RRs are available in Table 3.

**Table 3:** Adapted BRIDGE criteria applied to 2016 rapid review reports

Criteria	All (***n*** = 103)	Journal published (***n*** = 52)	Non-journal published (***n*** = 51)	OR (95% CI)	***P*** value^a^
		***n*** (%)	***n*** (%)		
A. RR commissioned or conducted for decision-making purposes	79 (77)	34 (65)	45 (88)	**0.26 (0.09–0.74)**	**0.01**
B. RR conducted through a rapid response service	21 (20)	1 (2)	20 (39)	**0.03 (0.00–0.20)**	**< 0.0001**
C. Topic identified through a priority-setting exercise	1 (1)	0 (0)	1 (2)	0.00 (0.00–18.63)	0.50
D. RR addresses					
Political and/or health systems contexts	63 (61)	30 (58)	33 (65)	0.75 (0.32–1.69)	0.55
Problem related to the issue	99 (96)	52 (100)	47 (92)	OR not available	0.06
Options	0 (0)	0 (0)	0 (0)	OR not available	1.00
Implementation considerations	32 (31)	15 (29)	17 (33)	0.81 (0.35–1.95)	0.67
Cost implications	36 (35)	13 (25)	23 (45)	**0.41 (0.17–0.98)**	**0.04**
RR addressed at least four or more of the above issues	14 (14)	6 (12)	8 (16)	0.70 (0.22–2.35)	0.58
E. RR attempted to synthesise research evidence	103 (100)	52 (100)	51 (100)	OR not available	1.00
F. RR incorporates tacit knowledge of policy-makers/stakeholders	33 (32)	15 (29)	18 (35)	0.75 (0.32–1.83)	0.54
G. If yes, knowledge collected in systematic, transparent way^b^	16 (48) (*n* = 33)	11 (73) (*n* = 15)	5 (28) (*n* = 18)	**6.67 (1.42–33.76)**	**0.01**
H. RR explicitly targets policy-makers and/or stakeholders	68 (66)	27 (52)	41 (80)	**0.27 (0.11–0.67)**	**0.003**
I. RR was reviewed by policy-makers and/or key stakeholders for relevance and clarity	28 (27)	10 (19)	18 (35)	0.44 (0.17–1.08)	0.08
J. RR reviewed by patients/consumers for relevance and clarity	6 (6)	3 (6)	3 (6)	0.98 (0.17–5.67)	1.00
K. RR formally involved patients in phases of the RR conduct	6 (6)	3 (6)	3 (6)	0.98 (0.17–5.67)	1.00
Across any of following phases:					
Preparatory phase	3	1	2		
Execution phase	1	1	0		
Translation phase	5	2	3		
L. RR organised to highlight decision-relevant information anywhere in the document^c^	27 (26)	6 (12)	21 (41)	**0.19 (0.07–0.53)**	**0.001**
	***Mean (SD)***			***MD (SE)***	***P value***
M. RR written in understandable/lay language					
Readability: SMOG Index (years of education)					
Abstract/Summary	13.97 (1.51)	13.91 (1.55)	14.24 (1.36)	−0.33 (0.29)	0.25
Introduction/Background	13.80 (1.75)	14.01 (1.91)	13.57 (1.55)	0.44 (0.34)	0.20
Discussions/Conclusions	14.03 (1.98)	13.79 (1.68)	14.35 (2.29)	−0.56 (0.40)	0.16
Word count					
Main body of the report	8471 (7196)	6708 (4575)	10,269 (8818)	**− 3561 (1388)**	**0.01**
Total word count (including references and appendices)	13,834 (13,382)	10,343 (10,051)	17,393 (15,385)	**− 7050 (2566)**	**0.01**
Reading time (minutes)					
Main body of the report	42 (36)	33 (23)	51 (44)	**−18 (6.94)**	**0.01**
Total report (all pages)	69 (67)	52 (50)	87 (77)	**−35 (12.82**)	**0.01**
N. RR prepared in a format that makes the information easy to absorb					
Yes, graded entry^d^	24 (23)	0 (0)	24 (47)	**0.00 (0.00–0.10)**	**< 0.0001**
Traditional IMRaD^e^	52 (50)	48 (92)	4 (8)	**125.49 (28.88–586.53)**	**< 0.0001**
Graded entry front end followed by IMRaD^f^	13 (13)	2 (4)	11 (22)	**0.15 (0.02–0.68)**	**0.01**
Multicomponent report^g^	14 (14)	2 (4)	12 (24)	**0.13 (0.02–0.59)**	**0.004**
O. RR findings contextualised through online commentaries/briefings provided by policy-makers and/or key stakeholders	5 (5)	3 (6)	2 (4)	1.49 (0.22–12.50)	1.00
P. RR brought to the attention of target audiences through email, listservs, public website posting	6 (6)	2 (4)	4 (8)	0.47 (0.06–2.67)	0.44
Q. RR addresses equity considerations	34 (33)	14 (27)	20 (39)	0.57 (0.24–1.38)	0.21
R. RR conveys formal recommendations	25 (24)	11 (21)	14 (27)	0.71 (0.29–1.86)	0.50
S. Methods to conduct the RR described	94 (91)	51 (98)	43 (84)	**9.32 (1.31–211.38)**	**0.02**
T. Quality assessment/risk of bias assessment of included studies	58 (56)	26 (50)	32 (63)	0.60 (0.26–1.31)	0.23
U. Limitations of the RR process or approach outlined/provided	29 (28)	24 (46)	5 (10)	**7.72 (2.62–23.47)**	**< 0.0001**
V. Reference of included studies provided	103 (100)	52 (100)	51 (100)	Not estimable	1.00
W. Local applicability discussed	55 (53)	19 (37)	36 (71)	**0.24 (0.10–0.56)**	**0.001**
X. Case examples included to illustrate how to adapt or apply the intervention/policy locally					
Yes	3	0	3	0.00 (0.00–1.66)	0.12
Not applicable (non-interventional RR)	11	10	1		
Y. Key messages or summary points provided	26 (25)	8 (15)	18 (35)	**0.34 (0.13–0.88)**	**0.02**
Z. Clearly labelled as ‘rapid’ (explicit phrasing or derivative)					
Yes, ‘rapid’ stated in the title	35 (34)	29 (56)	6 (12)	**9.23 (3.42–25.79)**	**< 0.0001**
If not stated in title, term labelled in the abstract/elsewhere in report	36 (35)	17 (33)	19 (37)		
Other term used to indicate abbreviated/timely (e.g. targeted review, mini-systematic)	19 (18)	4 (8)	15 (29)		
Non-descript label used (e.g. evidence note, evidence summary)	13 (13)	2 (4)	11 (22)		
Rapid review terminology consistently used to describe the report^h^	73 (71)	35 (67)	38 (75)		

*OR* odds ratio, *CI* confidence interval, *SD* standard deviation, *MD* mean difference, *SE* standard error

^a^*P* value based on Fisher’s Exact Test for binomial counts or Welch’s *t* test for continuous score

^b^Systematic collection may include, for example, formal feedback from an expert panel or working group; through surveys, key informant interviews, or Delphi process

^c^Reviewers were asked of the report need to fish around the report in order to pull out key information to make a decision or what this information easily identified in the report?

^d^Graded entry is a report format organised to highlight decision-relevant, summarised information upfront with access to additional, more in-depth information

^e^IMRaD: a report format structured to include the following sections consecutively: Introduction, Methods, Results and Discussion sections of an original article

^f^Graded entry plus IMRaD refers to a document that combines a graded entry front end followed by a structure that includes the various IMRaD components

^g^Multicomponent report refers to a report divided into various ‘chapters’ or ‘sections’ beyond the typical IMRaD or general graded entry structures

^h^Reports using inconsistent terminology include those, for example, that use the term ‘rapid’ but also label as ‘systematic review’ somewhere in the report

#### What was covered

A large portion of the RRs (77%; *n* = 79) were reportedly commissioned or produced for decision-making purposes. Fewer (20%; *n* = 21) were conducted as part of a rapid response service while only one RR was part of a priority-setting exercise used to guide the focus of another SR. Most RRs (96%; *n* = 99) described a problem or the issue at hand, while a large segment of the RRs (61%; *n* = 63) addressed aspects of political and/or health systems context. Cost implications (35%; *n* = 36) and implementation considerations (31%; *n* = 32) were covered by a lesser proportion of the RRs. None outlined possible options to address policy, treatment or implementation.

#### What was included

By virtue of the fact that the information products being assessed in this case were all RRs, every report was deemed to have provided a level of research evidence synthesis. We further assessed that nearly a third of the RRs (32%; *n* = 33) involved the tacit knowledge of policy-makers or stakeholders in the process in some capacity, for which this knowledge was collected in a systematic and transparent way in nearly half of these instances (48%; *n* = 16). Type of involvement included, for example, establishing formal advisory or working groups, round table policy discussions, the use of semi-structured interviews, key informant interviews and use of a Delphi method.

#### For whom its targeted

The majority of RRs (66%; *n* = 68) seemed to target policy-makers and key stakeholders as the intended audience but fewer (27%; *n* = 28) reported to engage with them directly to discuss and review the content of the RRs for relevance and clarity. Further, only six RRs (6%) were reviewed by patients or consumers for content and clarity. This mostly included patient/partner involvement in dissemination of the report versus planning or conducting the review.

#### How it is packaged

Only 26% (*n* = 27) of RRs were organised to highlight decision-relevant information anywhere in the report. Less than a quarter of the RRs (23%; *n* = 24) used a graded entry format that decision-makers could easily scan for pertinent information. Most RRs were structured according to the traditional IMRaD approach (50%; *n* = 52), a graded entry front end with the remainder of the report in IMRaD format (13%; *n* = 13) or a lengthier, multicomponent report format (14%; *n* = 14). Additionally, based on the word counts for each RR, the average reading time of the main body of reports was a mean (standard deviation) of 42 (36) minutes. Further, we assessed the reading level a person would need in order to understand the text of the RRs easily on first reading. SMOG scores of the abstract/summary, introduction/background and discussion/conclusion sections were 13.97, 13.80 and 14.03, respectively, corresponding to the years of formal education past the age of six needed to understand the text across these sections.

#### How its use is supported

Only five RRs (5%) reported that policy-makers or stakeholders had provided online contextualisation or briefings. Similarly, six RRs (6%) reported disseminating report findings by targeting key stakeholders through email, listservs or through website postings.

#### Features and content

Equity considerations were discussed or implicitly considered by the nature of the topic in one-third of the RRs (33%; *n* = 34). Nearly one-quarter of the RRs (24%; *n* = 25) stated formal recommendations. A high proportion of RRs described the methods employed (91%; *n* = 94) and all RRs provided a reference list of included studies (100%; *n* = 103). Several RRs involved quality assessment of the included studies (56%; *n* = 58), while reference to limitations of the RR process as compared to a traditional SR (28%; *n* = 29) or providing a specifically labelled list of key messages or summary points (25%; *n* = 26) was less common. Although local applicability was discussed to some degree in several of the RRs (53%; *n* = 55), only three RRs included specific case examples to illustrate how to apply or adapt a policy or intervention locally.

Collectively, the majority of RRs (69%; *n* = 71) explicitly used the term ‘rapid’ in the title (34%; *n* = 35) or in the abstract or elsewhere in the document (35%; *n* = 36). However, other terms implying rapid or abbreviated (e.g. targeted review, mini-systematic review) were also identified in a portion of the RRs (18%; *n* = 19). For some RRs (13%; *n* = 13), there was no indication of the term ‘rapid’ in the labelling as non-descript terms were used (e.g. evidence summary, evidence note) yet the methods reflected a RR approach. Further, for a majority of RRs (71%; *n* = 73) there was consistent labelling used within reports.

#### Exploratory analysis of JP versus NJP rapid reviews

This analysis revealed that, for certain items, there were differences noted between JP and NJP RRs (Table 3). For example, although a similar number of RRs incorporated the tacit knowledge of policy-makers and stakeholders in the process across both groups (Item F), a greater number of JP RRs collected this knowledge in a systematic and transparent way (Item G) (JP 73% vs. NJP 28%; OR 6.67, 95% confidence interval (CI) 1.42–33.76). In addition, we also observed a higher percentage of JP RRs meeting additional criteria as compared to the NJP RRs, including using an IMRaD format (JP 92% vs. NJP 8%; OR 125.49, 95% CI 28.88–586.53); providing a description of the methods used to conduct the reviews (Item S) (JP 98% vs. NJP 84%; OR 9.32, 95% CI 1.31–211.38); stating the limitations of the RR approach or process (Item U) (JP 46% vs. NJP 10%; OR 7.72, 95% CI 2.62–23.47); and declaring the review as ‘rapid’ in the title (Item Z) (JP 56% vs. NJP 12%; OR 9.23 (95% CI 3.42–25.79).

With regards to the NJP RRs, certain criteria were found to be proportionately higher in comparison to JP RRs (Table 3). This included a higher percentage of RRs commissioned or conducted for decision-making purposes (Item A) (JP 65% vs. NJP 88%; OR 0.26, 95% CI 0.09–0.74) and RRs conducted through a rapid response service (Item B) (JP 2% vs. NJP 39%; OR 0.03, 95% CI 0.00–0.20). Further, the NJP RRs were more likely to have addressed cost implications (Item D) (JP 25% vs. NJP 45%; OR 0.41, 95% CI 0.17–0.98) and explicitly targeted policy-makers and key stakeholders (Item H) (JP 52% vs. NJP 80%; OR 0.27, 95% CI 0.11–0.67). In addition, a higher proportion of NJP RRs were organised to highlight decision-relevant information (Item L) (JP 12% vs. NJP 41%; OR 0.19, 95% CI 0.07–0.53) and used a graded entry format (JP 0% vs. NJP 47%; OR 0.00, 95% CI 0.00–0.10), graded entry plus IMRaD format (JP 4% vs. NJP 22%; OR 0.15, 95% CI 0.02–0.68), or were integrated into a multi-component report (Item N) (JP 4% vs. NJP 24%; OR 0.13, 95% CI 0.02–0.59). Further, a greater number of NJP RRs made reference to local applicability (Item W) (JP 37% vs. NJP 71%; OR 0.24, 95% CI 0.10–0.56) and presented key messages or summary points for the end-users (Item Y) (JP 15% vs. NJP 35%; OR 0.34, 95% CI 0.13–0.88). In addition, RRs that were NJP had significantly higher word counts for both the main body of the report and when assessing the entire document. Therefore, it follows that reading time was also significantly longer for these RRs (i.e. on average 18 minutes longer to read, JP 33 minutes vs. NJP 51 minutes) (Item M – Main body of the report). In terms of labelling (Item Z), NJP RRs were more likely to use non-descript labels (JP 4% vs. NJP 22%) or alternate terms to ‘rapid’ more often to indicate timely or abbreviated methods (JP 8% vs. NJP 29%).

## Discussion

Evaluating the extent to which RRs do in fact help bridge the gap between evidence research and policy is important. Applying the modified BRIDGE criteria to our sample, we were able to do an initial assessment of RRs as an information-packaging mechanism intended to gather relevant evidence in one place, to provide contextualised information for a current region or jurisdiction, and to make health information easier to understand and use. Overall, conformity with the BRIDGE criteria was modest. Further, findings suggest that many of the RRs identified had several useful features when examined against the criteria but also highlight areas for potential improvement (Box 1).

Across criteria, the majority of RRs were judged to have been commissioned or undertaken specifically for decision-making purposes and were therefore deemed to be topical or focused on issues of relevance to policy-makers and key stakeholders. However, as a collective, it did not appear to be common practice to use an explicit process of determining topic relevancy (i.e. using a rapid response service or priority-setting exercise to determine the topic), although a closer look showed that NJP RRs were more apt to have come through a response service as compared to JP RRs. Rapid response-type services run by experienced reviewers, through the totality of the intake process, should include discussions between the requestor and the review team, and lead to identification and refinement of answerable questions, and understanding of priority and feasibility to best meet information needs. Further, specific priority-setting exercises should be considered for those stakeholder groups that have competing topics in need of review. The practicalities of producing timely evidence should be aligned with the need for a timely decision and/or rapid implementation and be included as part of priority-setting plans.

As outlined in the criteria, information-packaging mechanisms should address the many features of the issue being covered. Describing the underlying problem or objectives of each review and including information on related political or health system contexts was well covered by this sample. However, cost implications and implementation considerations were addressed less often and none of the RRs referred to options for addressing the underlying problem or other ways to achieve the objectives of the stated issue. RR producers, through dialogue with requestors or commissioners of RRs, at the outset should ensure this information is solicited and incorporated into the report as part of contextual information provided in the background and integrated into the rationale presented for doing the RR. Recently, the SelecTing Approaches for Rapid Reviews (STARR) tool was developed to aid review authors in planning approaches when conducting RRs [46]. Importantly, it emphasises a shared understanding between RR teams and commissioners and clear communication to ensure a common awareness as to the purpose and context of the RR, questions to be answered, and how the review will be conducted and used.

Although a large portion of the identified RRs targeted healthcare policy-makers or specific stakeholders, only one-third formally incorporated the tacit knowledge of these end-users into the RR process. Of those that did, few collected and reported such knowledge in a systematic and transparent manner. In addition, policy-makers or key stakeholders were involved in reviewing less than one-third of the RR draft reports or manuscripts. Going forward, those producing RRs for decision-making purposes should give consideration as to how best to elicit tacit as well as explicit knowledge using open communication and conversation directly with stakeholders as engagement serves to enhance the relevance and applicability of the reviews in the decision-making process [47, 48]. Based on existing guidance, the level of engagement should be meaningful, yet designed in accordance with available resources with partnerships established early in RR the process [49].

Patients should also be recognised as relevant knowledge users and benefactors of research evidence stemming from RRs. Therefore, we modified the BRIDGE criteria to capture patient engagement, which findings indicate is minimal across the RRs. Although not a new concept, patient-oriented research is often overlooked in large part because researchers lack guidance and promising practices on how to effectively engage patients and their families in designing and conducting research [50]. To date, patient/partner involvement in knowledge synthesis has been limited despite the demonstrated success of how patients can play a role in the production of SRs [51]. By extension, we need to find innovative ways to feasibly involve patients in the planning, conduct and knowledge translation of RRs.

When we examined how RRs are packaged, roughly one-quarter of our sample were judged as organised in some manner to highlight decision-relevant information, including, for example, benefits and harms, costs of policy or programme options. Most often, this information was not easily identifiable and required searching through various sections of text to locate. Key messages or summary points were also provided in only one-quarter of our sample. Further, only 23% of our sample was prepared in a format that makes the information easy to absorb (i.e. graded entry), while 50% were prepared using the standard publishing format used in academic journal articles (i.e. IMRaD) [41]. Although several studies indicate that policy-makers are more partial to the graded entry format [42, 52, 53], a recent study showed that, while policy-makers favoured an alternative order to IMRaD, healthcare managers preferred a more conventional ordering of information [54]. Therefore, further research is needed to determine which report structures are perceived as most useful and for which end-users and, importantly, which formats result in better comprehension and uptake of RR findings. At the moment, it is not known how formats and features, subject matter of the reviews, and individual factors intersect to impact the use of RRs.

Cursory assessment of readability suggests that, as a collective, the packaging of RRs for stakeholders could also be improved if documents were more succinct (i.e. took less time to read) and were clearly written in plain language so that end-users are able to make the most sense of the evidence they examine [27, 55, 56]. The written content of the RRs (i.e. requiring approximately 13–14 years of formal schooling to comprehend the text) is quite complex and equates to a university reading level [38]. Although there are no reading level standards specific for healthcare professionals, including policy-makers, in order to reach people with low levels of literacy, research suggests that written health materials should be aimed at Grade 8 or below in the United States and Grade level 12 in the United Kingdom [57]. The lesson from this study is that RR producers should aim to reduce writing complexity as much as possible without being overly simplistic so readers will comprehend and retain ideas more reliably. We caution that a more comprehensive evaluation of the text of RRs is needed and should involve other readability measures and assess additional factors such as reading time, amount recalled and overall comprehension.

In terms of better supporting the use of RRs, producers and commissioners should consider mechanisms by which concise online commentaries or briefings could be provided by the policy or stakeholder leaders that the RRs were intended to target (e.g. AHRQ Views). In addition, efforts to disseminate findings to key audiences using various communication channels, for example, email, listservs, websites and blog posts, should be considered. Social media platforms also offer the potential to promote RR evidence.

As for additional features and content, we found that 44% of our sample did not include quality assessment or risk of bias of the included studies, which is less than previously reported [7]. Part of clearly communicating research findings to end-users is providing an accurate overall assessment of research underpinning the topic or intervention being reviewed. This means that each included study in a RR, to the extent possible, should be critically appraised and include an assessment of key sources of bias. Providing limitations of the evidence (e.g. risk of bias, publication bias) at the study level should be described in order to help interpret overall confidence in the results, as is done when conducting SRs.

RR authors should also be encouraged to highlight potential sources of bias introduced into the RR process itself, depending on the abbreviated methods used as well as any other methodological concerns. However, less than half of the RRs in our sample outlined such limitations. Although there is no instrument specific to RRs to assess the quality of conduct or bias, with some adjustments, AMSTAR-2 [58] and ROBIS [59] could both be applied to assess the methodological restrictions compared to a SR, risk of bias and validity of the results. In addition, a reporting guideline extension for RRs, currently under development [60], will be a useful tool for researchers to improve accuracy, completeness and transparency of reporting.

The exploratory analysis showed that several differences between JP and NJP RRs are likely due to the nature of academic journal publishing that stipulates the format, type and length of the content presented in articles. For example, JP RRs were shorter in length, more often described review methods and acknowledged the limitations of the process. Conversely, NJP RRs are produced by organisations, with varying mandates, that can freely design and tailor RR products for various knowledge-user audiences. Paradoxically, this autonomy may not always facilitate better use of RRs for end-users, for example, if they are lengthier to read. However, more often, NJP RRs were organised to highlight key messages and decision-relevant information using non-traditional report formats to convey findings. Ideally, the best features from each publication type should be combined to inform best practices and future recommendations for how RRs are packaged. The needs and preferences of different end-users (e.g. policy-makers, clinicians, health systems managers, researchers) should also be evaluated and considered in further shaping RRs as an information product. Currently, we have little knowledge about the specific target audiences for the JP and NJP RRs and whether they vary across publication types and, if so, to what extent. It, too, requires further research and exploration.

### Limitations

For most items, we judged ‘yes’ or ‘no’ as to whether an item was met but did not assess how well items were reported in the RRs as this was beyond the scope of our study. Although the original authors of the BRIDGE criteria openly encouraged its further adaptation, we may not have interpreted the previous criteria in the same manner as was originally intended, as modifications made to the criteria were meant to align with the context of producing RRs to inform decision-making in healthcare. Nonetheless, future studies involving RRs should explore both the face and content validity of these items with a variety of stakeholder groups. An additional limitation of our study was that we restricted our sample to only those RRs produced in 2016 in English or French due to resource limitations. It is important to acknowledge that there are many productive RR initiatives from various regions around the globe that produce RRs in other languages (e.g. Portuguese, Spanish, German), which are not reflected in our findings. Therefore, we recognise our sample is not representative of the entire population of RRs. However, we did aim to increase the generalisability of our results by including a heterogeneous group of RRs produced in various countries.

We also recognise that some of the BRIDGE criteria may not apply to all RRs depending on their purpose or intended use, the topic under review, and the degree of tailoring involved. For example, some RRs may present and aid interpretation of the evidence only rather than provide formal recommendations as the criteria suggest. Another example is that not all RRs are publicly available due to proprietary reasons or require a fee or subscription to access them from the producer. Therefore, support of their use publicly through online commentaries, website posting, emails or listservs would not be allowed and, consequently, related BRIDGE criteria not applicable. Last, we acknowledge the potential issue of multiple testing related to exploratory analyses and often unknown inflation of the alpha-level with selective reporting of tests and their impact on *P* values. However, as laid out in our protocol, our exploratory analysis was planned and carried out as documented.

## Conclusions

Findings suggest that, of the 103 RRs assessed, adherence to the modified BRIDGE criteria was modest. Many RRs had several useful features when examined against these criteria for communicating clearly and document features recognised to be valued by end-users of research. However, there were several RRs for which elements of the modified BRIDGE criteria were not well demonstrated or lacking and that represent areas for potential improvement. Our research findings fill an information gap related to the suitability and usability of RRs as a knowledge translation product. Moreover, for producers of future RRs, including those produced by new or existing rapid response services around the world, these findings highlight potential implications regarding a range of operational, content and design elements for consideration when undertaking RRs. Importantly, the packaging of information in RRs is relevant and, ideally, should best meet the information needs of policy-makers and key stakeholders to optimise the uptake of evidence from RRs in healthcare decision-making.

## Contributions to the literature

This study is novel in that it is the first to assess RRs as an information product; namely, how well they are parcelled for use in decision-making for policy-makers and other stakeholders. This study is also intended to help guide researchers who want to communicate their RR findings more effectively so that decision-makers can make use of the best available health research evidence. Importantly, this work is intended to promote innovation in how future RRs are reported and packaged and encourages the importance of key healthcare stakeholders being involved in their future development.

Box 1 Potential areas for improvements to better meet the information needs for policy-makers and other stakeholders ▪ Use an explicit process (i.e. a rapid response service and/or priority-setting exercise) to determine relevant and priority topics from the perspective of the policy-maker or other stakeholders

▪ Consider information on cost implications and implementation considerations as well as options for addressing the underlying problem or objectives of the stated issue being reviewed

▪ Include the tacit knowledge of policy-makers and other stakeholders in the rapid review (RR) process

▪ Provide as assessment of methodological quality/risk of bias of the included studies to aid in the interpretation of findings and confidence in the results of the RR

▪ Address equity considerations

▪ Address local applicability by placing evidence in context

▪ Involve policy-makers and other stakeholders in review of draft reports or manuscripts to improve relevance and clarity

▪ Consider ways to involve patients as relevant knowledge users of RRs

▪ Organise RRs to highlight decision-relevant information (e.g. benefits and harms, costs of policy or programme options)

▪ Design RR reports so that information is easy to absorb (i.e. use a graded entry report format)

▪ Prepare RRs that are succinct and are clearly written in plain language so they are easily read and understood

▪ Contextualise the RR through online commentaries/briefings provided by policy-makers or stakeholders

▪ Consider various communication channels to disseminate findings to key audiences

▪ Provide clear consistent labelling of RR products

## Supplementary information

**Additional file 1.** STROBE Statement — Checklist of items that should be included in reports of cross-sectional studies.

**Additional file 2.** Journal characteristics of the journal-published rapid reviews (2016).

## Data Availability

The dataset(s) supporting the conclusions of this article is (are) included within the article (and its additional file(s)).

## Abbreviations

CI: confidence interval
IMRaD: introduction, methods, results, and discussion
JP: journal published
NJP: non-journal published
OR: odds ratio
RR: rapid review
SMOG: Simple Measure of Gobbledygook
SR: systematic review
