# Supplementary material for: Assessing how information is packaged in rapid reviews for policy-makers and other stakeholders: a cross-sectional study
Source: Health Res Policy Syst. 2020 Sep 29;18:112. doi: 10.1186/s12961-020-00624-7 (PMC7523380; doi:10.1186/s12961-020-00624-7)
Supplement: Supplementary file 1 — Additional file 1. STROBE Statement — Checklist of items that should be included in reports of cross-sectional studies. [file 12961_2020_624_MOESM1_ESM.pdf]

Additional File 1. STROBE Statement—Checklist of items that should be included in reports of *cross-sectional studies*

NOTE: There is no guideline specific to this type of methodological study. Therefore, we have used guidelines for reporting observational research as a proxy to ensure all relevant reporting has been included in the final manuscript. Specifically, we have followed the STROBE statement (for cross sectional studies) as a guide. Given this, we note that certain aspects of our research may not align and therefore, not comply fully with the pre-specified checklist. Nonetheless, key elements of conduct have been reported on fully and transparently within the confines of the manuscript and appendices.

	Item No	Recommendation	Reported on Page Number (#)
**Title and abstract**	1	(*a*) Indicate the study's design with a commonly used term in the title or the abstract	Pg. 1 – stated directly in the title
		(*b*) Provide in the abstract an informative and balanced summary of what was done and what was found	Pg. 2
**Introduction**			
Background/rationale	2	Explain the scientific background and rationale for the investigation being reported	Pgs. 4-5
Objectives	3	State specific objectives, including any prespecified hypotheses	Pg. 5
**Methods**			
Study design	4	Present key elements of study design early in the paper	Pg. 6 Methods section
Setting	5	Describe the setting, locations, and relevant dates, including periods of recruitment, exposure, follow-up, and data collection	Pgs. 6-11 Methods details: unit of analysis - rapid review (RR) reports
Participants	6	(*a*) Give the eligibility criteria, and the sources and methods of selection of participants	Pgs. 6-7 Details on how data set was derived (unit of analysis is the RR). Also, Figure 1 provides information on study flow (included/excluded studies)
Variables	7	Clearly define all outcomes, exposures, predictors, potential confounders, and effect modifiers. Give diagnostic criteria, if applicable	Pgs. 7-9 – details on modified BRIDGES criteria provide. Information also provided in Tables 1 & 3 and Figures 2 &3. Also, general study characteristics captured – noted in Table 2.
Data sources/ measurement	8*	For each variable of interest, give sources of data and details of methods of assessment (measurement). Describe comparability of assessment methods if there is more than one group	*n/a*

Bias	9	Describe any efforts to address potential sources of bias	Study limitations referred to on pgs. 20-21.
Study size	10	Explain how the study size was arrived at	Pgs. 6-8
Quantitative variables	11	Explain how quantitative variables were handled in the analyses. If applicable, describe which groupings were chosen and why	Pgs. 10-11
Statistical methods	12	(*a*) Describe all statistical methods, including those used to control for confounding	Pg. 10-11 describes statistical methods applied. We did not apply statistical methods to control for confounding for this methods study.
		(*b*) Describe any methods used to examine subgroups and interactions	n/a
		(*c*) Explain how missing data were addressed	n/a
		(*d*) If applicable, describe analytical methods taking account of sampling strategy	n/a
		(*e*) Describe any sensitivity analyses	n/a
**Results**			
Participants	13*	(a) Report numbers of individuals at each stage of study—eg numbers potentially eligible, examined for eligibility, confirmed eligible, included in the study, completing follow-up, and analysed	See Results section – pgs. 11-12 and Figure 1 – study flow diagram.
		(b) Give reasons for non-participation at each stage	See Figure 1; Results pg. 11-12
		(c) Consider use of a flow diagram	See Figure 1
Descriptive data	14*	(a) Give characteristics of study participants (eg demographic, clinical, social) and information on exposures and potential confounders	Pg. 11-12; Table 2 (characteristics of included studies)
		(b) Indicate number of participants with missing data for each variable of interest	See Table 3
Outcome data	15*	Report numbers of outcome events or summary measures	See Table 3 and Figures 2 & 3. Also, Results Section – pgs. 1-16
Main results	16	(*a*) Give unadjusted estimates and, if applicable, confounder-adjusted estimates and their precision (eg, 95% confidence interval). Make clear which confounders were adjusted for and why they were included	For the exploratory analysis only – see page 16 & Table 3 (as reported in the methods section; estimated associations were crude and based on a univariate analysis and therefore, were not adjusted for other factors)
		(*b*) Report category boundaries when continuous variables were categorized	n/a

		(*c*) If relevant, consider translating estimates of relative risk into absolute risk for a meaningful time period	n/a
Other analyses	17	Report other analyses done—eg analyses of subgroups and interactions, and sensitivity analyses	As per Methods Section – we conducted an exploratory analysis to assess potential differences for items between journal published RRs and non-journal published RRs (See Table 3 for full results)
**Discussion**			
Key results	18	Summarise key results with reference to study objectives	Pgs. 10-16 – Results; Discussion section – key points summarized – pgs. 16-20; Conclusions Pg. 21
Limitations	19	Discuss limitations of the study, taking into account sources of potential bias or imprecision. Discuss both direction and magnitude of any potential bias	Pgs. 20-21
Interpretation	20	Give a cautious overall interpretation of results considering objectives, limitations, multiplicity of analyses, results from similar studies, and other relevant evidence	Pgs. 16-21
Generalisability	21	Discuss the generalisability (external validity) of the study results	Pg. 20 – Strengths & Limitations section
**Other information**			
Funding	22	Give the source of funding and the role of the funders for the present study and, if applicable, for the original study on which the present article is based	Pg. 22
