# Supplementary material for: Assessing how information is packaged in rapid reviews for policy-makers and other stakeholders: a cross-sectional study
Source: Health Res Policy Syst. 2020 Sep 29;18:112. doi: 10.1186/s12961-020-00624-7 (PMC7523380; doi:10.1186/s12961-020-00624-7)
Supplement: Supplementary file 2 — Additional file 2. Journal characteristics of the journal-published rapid reviews (2016). [file 12961_2020_624_MOESM2_ESM.pdf]

Additional File 2. Journal Characteristics of the Journal Published Rapid Reviews (2016)

Unique Journals (n=47)	Publisher	Peer Review	Journal Impact Factor (IF)
Human Resources for Health	BioMed Central (BMC)	✓	1.780
Infectious Diseases of Poverty	BioMed Central (BMC)	✓	3.181
Systematic Reviews	BioMed Central (BMC)	✓	1.052
BMC Health Services Research	BMC	✓	1.827
Emergency Medicine Journal	BMJ Publishing Group	✓	1.861
Arch Dis Child	BMJ Publishing Group	✓	3.265
BMJ Open	BMJ Publishing Group	✓	2.369
Australian Journal of Primary Health	CISRO Publishing	✓	1.246
Int. J. Nurs. Educ. Scholarsh.	De Gruyter	✓	No IF
Appetite	Elsevier	✓	3.403
Child Abuse & Neglect	Elsevier	✓	2.293
Clinical Neurology and Neurosurgery	Elsevier	✓	1.381
Int. J. Oral Maxillofac. Surg.	Elsevier	✓	1.918
Journal of Emergency Medicine	Elsevier	✓	3.214
Journal of the American Dental Association	Elsevier	✓	1.998
Public Health	Elsevier	✓	0.853
The Breast	Elsevier	✓	1.210
International Journal of Prisoner Health	Emerald Insight	✓	No IF
Ontario Health Technology Assessment Series	Health Quality Ontario (HQO)	×	0.919
Can J Gastroenterol Hepatol.	Hindawi Publishing Corp.	✓	2.147
Canadian Respiratory Journal	Hindawi Publishing Corp.	✓	1.153
Rural and Remote Health	James Cook University	✓	No IF
Caries Res	Karger Publishers	✓	1.811
Journal of Wound Care	MA Healthcare Limited, (Mark Allen Group)	✓	1.755
Health Services and Delivery Research	NIHR (UK)	✓	No IF
Health Technology Assessment	NIHR (UK)	✓	4.236
Gerontologist	Oxford Academic	✓	3.505
Health Promotion International	Oxford Academic	✓	1.722
Journal of Public Health	Oxford Academic	✓	No IF
Tropic Journal of Pharmaceutical Research	Pharmacotherapy Group, Faculty of Pharmacy, University of Benin, Nigeria	✓	0.569
PLoS Neglected Tropical Diseases	PLoS	✓	2.806
PLoS ONE	PLoS	✓	1.538
Australian & New Zealand Journal of Psychiatry	SAGE	✓	4.036
Canadian Journal of Occupational Therapy	SAGE	✓	1.255
European Journal of Cardiovascular Nursing	SAGE	✓	2.763
Journal of Medical Screening	Sage	✓	No IF
Journal of Primary Care & Community Health	Sage	✓	2.125
Journal of Research in Nursing	Sage	✓	1.446
Nursing Ethics	SAGE	✓	No IF
Perspectives in Public Health	SAGE	✓	3.834
Eur J Nucl Med Mol Imaging	Springer	✓	7.277
Teaching and Learning in Medicine	Taylor & Francis	✓	2.801
The Lancet	The Lancet	✓	47.831
Journal of Advanced Nursing	Wiley	✓	No IF
Journal of Evidence-Based Medicine	Wiley	✓	1.864
Transpl Infect Dis	Wiley	✓	1.719
Journal of Head Trauma Rehabilitation	Wolters Kluwer Health	✓	2.150
